# Assessment of safety and efficacy of mesenchymal stromal cell therapy in preclinical models of acute myocardial infarction: a systematic review protocol

**DOI:** 10.1186/s13643-017-0601-9

**Published:** 2017-11-07

**Authors:** Carly C. Barron, Manoj M. Lalu, Duncan J. Stewart, Dean Fergusson, Homer Yang, David Moher, Peter Liu, David Mazer, P. J. Devereaux, Lauralyn McIntyre, On Behalf of the Canadian Perioperative Anesthesia Clinical Trials Group

**Affiliations:** 1 Department of Anesthesiology and Pain Medicine, The Ottawa Hospital, Ottawa, Canada; 2 Blueprint Translational Research Group, Clinical Epidemiology Program, Ottawa Hospital Research Institute, Ottawa, Canada; 3 Department of Medicine, McMaster University, Hamilton, Canada; 4 Regenerative Medicine Program, Ottawa Hospital Research Institute, Ottawa, Canada; 5 Department of Epidemiology & Community Medicine, University of Ottawa, Ottawa, Canada; 6 Department of Cell and Molecular Medicine, University of Ottawa, Ottawa, Canada; 7 The Ottawa Heart Institute, Ottawa, Canada; 8 Department of Anesthesia, St. Michael’s Hospital, University of Toronto, Toronto, Canada; 9 Population Health Research Institute, David Braley Cardiac, Vascular, and Stroke Research Institute, McMaster University, Hamilton, Canada; 10 Clinical Epidemiology Program, Ottawa Hospital Research Institute, Department of Medicine (Division of Critical Care), University of Ottawa, 501 Smyth Rd, Box 201, Ottawa, ON K1H 8L6 Canada

**Keywords:** Mesenchymal stromal cells, Mesenchymal stem cells, Perioperative myocardial infarction, Myocardial infarction, Preclinical, Systematic review protocol

## Abstract

**Background:**

Despite advances in treatment, acute myocardial infarction (MI) is still associated with significant morbidity and mortality, especially in patients with extensive damage and scar formation. Based on some promising preclinical studies, there is interest in the use of mesenchymal stromal cells (MSCs) to promote cardiac repair after acute MI. However, there is a need for a systematic review of this evidence to summarize the efficacy and safety of MSCs in preclinical models of MI. This will better inform the translation of MSC therapy for acute MI and guide the design of a future clinical trial.

**Methods/design:**

A systematic literature search of MEDLINE, Embase, and BIOSIS Previews will be conducted. We will identify comparative preclinical studies (randomized and non-randomized) of myocardial infarction that include animals given MSC therapy versus a vehicle/placebo. The primary outcome will be left ventricular ejection fraction. Secondary and tertiary outcomes will include death, infarct size, measures of cardiac function, biochemical outcomes, and MSC retention and differentiation. Risk of bias will be assessed using the Cochrane Risk of Bias Tool. Subgroup analyses will be performed to measure how various sources of preclinical study heterogeneity affect the direction and magnitude of the primary outcome. We will meta-analyze data using inverse variance random effects modeling.

**Discussion:**

This systematic review of preclinical evidence will provide a summary of the efficacy and safety of MSCs in animal models of MI. The results will help determine whether sufficient evidence exists to conduct a clinical trial in humans and inform its design.

**Electronic supplementary material:**

The online version of this article (10.1186/s13643-017-0601-9) contains supplementary material, which is available to authorized users.

## Background

Cardiovascular disease is the leading cause of mortality in the western world [1]. Acute myocardial infarction (MI) can lead to permanent loss of cardiomyocytes and scar tissue formation and, in the event of a large area of injury, may result in heart failure and life-threatening arrhythmia. Despite advances in treatment such as coronary revascularization, some MI patients are left with extensive cardiac damage and a poor prognosis, highlighting the need to develop novel therapies to repair non-functional myocardium.

Over the past decade, mesenchymal stromal cells (MSCs)—also known as adult stem cells, marrow stromal cells, or mesenchymal stem cells—have emerged as a potential new therapy for acute MI. These cells can be isolated from a variety of tissues including bone marrow, adipose tissue, and the umbilical cord and (because they appear to be relatively immune privileged) can be subsequently delivered as an allogeneic product to patients [2]. In individual studies using preclinical models of acute MI, MSCs have been demonstrated to augment tissue repair, improve cardiac function [3], dampen the inflammatory response [4], and potentially reduce mortality [5]. However, these preclinical studies have not been systematically summarized to examine the efficacy of these cells in acute MI. Members of our group and others have demonstrated that MSC therapy acts via a myriad of paracrine pathways to dampen inflammation and augment cytoprotection [6–8]. Moreover, MSCs can improve cellular energetics by transferring mitochondria [9]. This is unlike drug-based therapeutics which largely act via “lock-and-key” mechanisms in which a specific substrate binds to a single active site matching its structure.

**Fig. 1:**
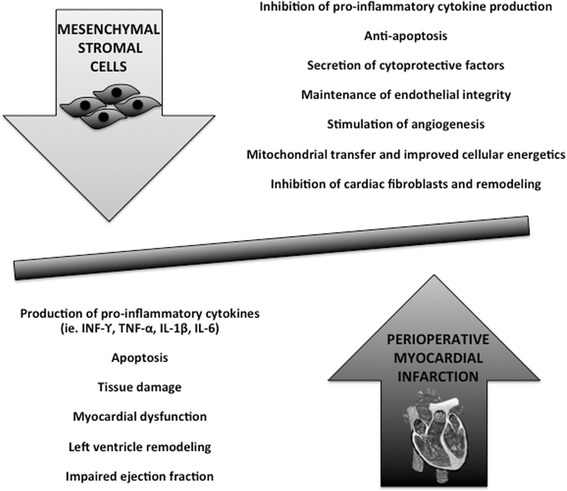
Therapeutic mechanisms of mesenchymal stromal cells in myocardial infarction

We are particularly interested in perioperative MI, which is an MI that occurs in the setting of inflammation and increased oxygen consumption induced by surgery [10, 11]. Perioperative MI is associated with poor outcomes, including a 30-day mortality of ~ 12% (vs. 2% for post-surgical patients without perioperative MI) [12]. Given the cytoprotective effects of MSCs, they may be particularly beneficial in the highly pro-inflammatory and catabolic setting of perioperative MI (see Fig. 1). Prior to considering a first-in-human clinical trial of MSC therapy for perioperative MI, we propose a comprehensive synthesis of the published literature. These data will determine whether additional evidence gaps remain to warrant further preclinical work, as well as future directions of MI research.

The specific aims of this systematic review are as follows:

1. To systematically compare the efficacy and safety of MSC therapy versus control in preclinical MI. Our primary outcome is left ventricular ejection fraction. Secondary endpoints include death, other measures of cardiac function, inflammatory markers, and vessel density. Tertiary endpoints will include cellular retention and differentiation.
2. Threats to internal validity will be evaluated using a modified version of the Cochrane Risk of Bias Tool for preclinical studies [13]. We will determine whether risk of bias influences the magnitude and direction of the primary endpoint.
3. External validity will be evaluated using subgroup analyses to measure how various sources of preclinical study heterogeneity (e.g., type of MI model, animal species, severity of MI) affect the direction and magnitude of the primary outcome.
4. Construct validity will be examined to evaluate the degree preclinical studies of MSC therapy for MI incorporate elements of clinical perioperative MI (e.g., pathophysiological elements), as this will be the focus of our future clinical trial.

## Methods and design

### Protocol

This systematic review protocol is reported in accordance with the Preferred Reporting Items for Systematic Review and Meta-Analysis Protocols (PRISMA-P) reporting guidelines [14]. A summary of the protocol will be listed on the Collaborative Approach to Meta-Analysis and Review of Animal Data from Experimental Studies (CAMARADES) website (http://www.camarades.info). The final review will be reported using the PRISMA guidelines [15].

### Data sources

We will search the following databases Ovid MEDLINE®, Ovid MEDLINE® In-Process & Other Non-Indexed Citations, Embase Classic + Embase, and BIOSIS. In addition, a manual review of the bibliographies of selected articles (e.g., reviews) will be performed.

### Search strategy

Search strategies will be developed by our research team in collaboration with an information specialist. Prior to final implementation, all strategies will undergo Peer Review of Electronic Search Strategies (PRESS) by another senior information specialist [16, 17]. Search strategies will use controlled vocabulary (e.g., Mesenchymal Stromal Cells) and keywords (e.g., MSCs) with adjustment for each database. We will apply preclinical filters to increase search efficiency [18–20]. Duplicate citations will be removed. The example search strategy (see Additional file 1) was used to search in MEDLINE.

### Eligibility criteria

Eligible studies include controlled comparative studies of preclinical MI or cardiac ischemia-reperfusion injury. We will include studies in which true randomization is performed using a method with a low risk of selection bias (such as computer random number generators and random number tables), as well as those that are quasi-randomized (i.e., by day of week or alternation) and non-randomized. This broad range of comparative studies will be included in order to answer our study question as terminology and methodology that is commonplace in clinical studies is not routinely employed in preclinical studies, and previous reviews have shown that randomization is reported in a third or less of animal studies [21]. Only peer-reviewed publications will be eligible with no restriction to publication year.

#### Population

We will include all preclinical in vivo models of experimentally induced MI that mimic pathophysiological aspects of clinical MI (see Table 1). Included studies will be perioperative (i.e., anesthetic provided before or concurrent to acute MI). In vitro studies, ex vivo studies, and neonatal MI models will be excluded.

**Table 1:** Preclinical models of perioperative myocardial infarction^a^

Class	Example
Ligation of the left coronary artery	Open chest, closed chest
Ischemia-reperfusion	Global ischemia-reperfusion model
Cryoinjury	Liquid nitrogen cooled copper probe used to injure coronary vessel
Microembolism	Injection of automicrothrombotic particulates into coronaries
Electrocauterisation	Direct electorcauterization of a coronary vessel
Pharmacological induction	Isoproterenol
Genetic model	Watanabe heritable hyperlipidemic rabbits with acute induced infarction

^a^All included models must provide an anesthetic either pre-induction or concurrent with the induction of myocardial infarction

#### Intervention

Studies using MSCs will be included; the International Society of Cellular Therapy consensus statement defining criteria for MSCs will be used as a guide [22]. We will include MSCs from xenogeneic, syngeneic, or allogeneic sources of any tissue origin. All delivery routes, including direct myocardial injection, intravenous and intra-arterial, will be considered. To be eligible, MSCs must be administered as a pretreatment or no later than 7 days following the induction of MI. This timing has been chosen to reflect the possible interventional window for a perioperative clinical trial.

Our focus will be on non-manipulated cells as this will be the intervention in a potential future trial. We will exclude differentiated MSCs (e.g., differentiated into a myocyte), genetically engineered MSCs, and MSCs administered by a scaffold system. Studies using MSCs only modified for cellular identification (e.g., reporter gene systems or nanoparticles) will be included. We will also exclude studies that investigate another novel agent as a co-treatment.

#### Comparator

All studies with a control arm of animals that have had experimental MI or cardiac ischemia-reperfusion injury (diseased control animals) induced and were treated with placebo/vehicle will be included.

### Outcomes

#### Primary endpoint

Left ventricular ejection fraction (LVEF), measured as a continuous variable at specific time points after MSC or control intervention, will be the primary endpoint. LVEF is a clinically meaningful endpoint since it has been linked to mortality following MI [23]. Physiologically, LVEF determines stroke volume, which together with heart rate determines cardiac output. It is also a feasible outcome as it is the most commonly reported cardiac function measure in preclinical studies [24, 25]. Various techniques are used to measure LVEF including two- or three-dimensional echocardiography, magnetic resonance imaging, and computed tomography. In our review, we will include and describe all techniques of LVEF measurement.

#### Secondary outcomes

Secondary outcomes will be a combination of dichotomous and continuous measures. A detailed listing of secondary/tertiary outcomes is provided in Table 2. Given the large number of outcomes, these results will be considered exploratory and interpreted cautiously. Secondary endpoints will include measures of cardiac function by echocardiography (e.g., cardiac output, fractional shortening, left ventricle end diastolic diameter, left ventricle end systolic diameter) and cardiac catheterization (e.g., left ventricular end diastolic pressure, left ventricular end systolic pressure, mean pulmonary artery pressure, right ventricular systolic pressure), biochemical outcomes (e.g., cytokines), infarct size, and vessel density. These measurements will provide additional support as to whether MSCs preserve ventricular function and prevent the pathological remodeling that occurs after MI. Furthermore, data on biochemical markers will help elucidate the role MSC therapy plays in regulating cellular and molecular mechanisms involved in the pro-inflammatory state following MI. Death will also be recorded; however, few studies use this endpoint due to considerations for animal welfare [26]. The occurrence of adverse events/negative effects with MSC administration will be recorded.

**Table 2:** *A priori* defined secondary and tertiary outcome measures

Outcomes	Comments and/or Examples of specific measures
Secondary outcomes:	
Death	Rarely used an outcome due to ethical concerns regarding animal welfare
Infarct size	Variety of quantifiable techniques (e.g. histological staining, nuclear imaging)
Cardiac function	Echocardiography
	◦ cardiac output
	◦ left ventricle end diastolic diameter
	◦ left ventricle end systolic diameter
	◦ fractional shortening
	Cardiac catheterization
	◦ cardiac output
	◦ left ventricular end diastolic pressure
	◦ left ventricular end systolic pressure
	◦ mean pulmonary artery pressure
	◦ right atrial pressure
Biochemical outcomes	Proinflammatory cytokines
	◦ interleukin-1beta, 6
	◦ tumour necrosis factor-alpha
	Anti-inflammatory cytokines
	◦ interleukin-10
	◦ transforming growth factor-beta1, 2, 3
Vessel density	Histological staining and quantification of vessels in cardiac tissue
Tertiary outcomes:	
Cellular retention	Imaging and quantification of labelled cells in host tissue
Cellular differentiation	Measurement of cardiac troponin in donor cells retained in host tissue

#### Tertiary outcomes

Tertiary endpoints will include MSC retention and differentiation (see Table 2). While our primary and secondary endpoints focus on measures that evaluate the efficacy of MSCs, the homing and potential differentiation of MSCs in myocardial tissue is also of interest.

#### Timing

The primary outcome of left ventricle ejection fraction and secondary biochemical outcomes and death will be collected at baseline, < 6 h, 6–24 h, > 24–72 h, > 72 h–1 week, > 1–3 weeks, > 3–4 weeks, and > 4 weeks after the administration of MSCs versus controls. These detailed intervals reflect the evolution of inflammation and remodeling in MI, described by our group and others [27–31]. In preclinical models of myocardial infarction, robust increases in expression of cytokines such as TNF-α, IL-1β, and IL-6 have been noted immediately after myocardial injury and up to 24 h later [32]. This is followed by a chronic remodeling phase in which cardiomyocytes are replaced by granulation tissue and a scar is formed at the infarct. Scar formation has been demonstrated by approximately day 14 post-infarct in mice, while a canine infarct is still evolving at this time point [29]. Therefore, our prespecified time intervals will capture outcomes during the post-infarct inflammatory response and repair of cardiac function in both small and large animal models of MI. All other secondary outcomes of cardiac function and tertiary outcomes of retention and engraftment will be collected at the latest time point, > 4 weeks after administration, to capture these measurements after the pro-inflammatory state and repair has occurred.

### Study selection and data extraction

Studies will be screened independently by two reviewers using dedicated cloud-based software (DistillerSR, Evidence Partners, Ottawa, Canada). Using the previously described a priori inclusion criteria, first level screening (title/abstract) will be liberal with both reviewers needed to exclude an article and one reviewer needed to include. We will be using an accelerated screening method for the title and abstracts in which the second reviewer will review records excluded by the first reviewer [33]. Second level screening (full study) will be performed independently in duplicate. If there are disagreements, the two individuals involved will review the case. If they cannot come to an agreement, a senior team member will provide the final decision. Reasons for exclusion will be recorded to enable a transparent selection process [34, 15].

Information from included studies will be collected on electronic data extraction forms. General categories include study characteristics (e.g., design), study population (e.g., species), MI model (e.g., cryoinjury), intervention and comparison (e.g., MSC dose), co-interventions (e.g., immunosuppressants, antibiotics, and cardiac medications), and preclinical outcomes (e.g., ejection fraction). Data extraction forms will be prepared a priori, and a calibration exercise will pilot five studies to refine the forms and ensure inter-rater consistency. Examples of data collection elements can be seen in Table 3.

**Table 3:** *A priori* defined data collection elements

Data collection element	Items
Study Characteristics	Author
	Year of publication
	Funding support
	Country
	Study design
	Total number of animals used
	N per independent intervention group
	Species
	Strain
	Gender
	Weight
	Mean age
	MI model (i.e. LAD ligation, ischemia reperfusion, cryoinjury, microembolism)
	Intercurrent illness of animal
	Anesthetic administered
Intervention Characteristics	Route of MSC delivery (intravenous, intracoronary, intramyocardial)
	Timing of MSC delivery
	Frequency of MSC delivery
	Source of MSCs (syngenic, allogeneic, xenogenic)
	Tissue origin of MSCs (bone marrow, adipose, Wharton’s jelly)
	Condition of MSCs (fresh, cryopreserved)
	Vehicle
	Defining criteria for MSCs

### Risk of bias assessment

As there is no validated tool to assess risk of bias in animal studies, we will describe potential biases using a modified version of The Cochrane Risk of Bias Assessment Tool [13]. Items include concealment of allocation, random sequence generation, blinding of personnel and endpoint measurements, and completeness of endpoint reporting. We will include additional domains relevant to animal studies such as source of funding, conflict of interest, sample size calculations, similarity of groups or adjustment for confounders at baseline, random housing of animals, and animal selection at random for outcome assessment. Risk of bias assessment will be carried out in duplicate by two independent reviewers. Disagreements will be resolved using the same process listed above. Each criterion will be assigned a value of low, high, or unclear risk of bias for each included study. A summary for all included studies will be presented in a table format. We have planned an analysis to determine the effects of high vs. low risk of bias on the effect size of the primary outcome.

### Assessment of external and construct validity

In preclinical studies, external validity describes the ability to generalize findings to different experimental conditions. External validity will be assessed by subgroup analysis of the primary outcome based on species, strain, age, sex, presence of intercurrent illness, MI model, ischemic time (if an ischemia-reperfusion model), MSC source (animal/tissue), timing of MSC administration (pretreatment vs. rescue) administration route, type of control, use of co-interventions (antibiotic, immunosuppressant, antihypertensive, statin, β-blocker, antiplatelet, anticoagulant therapies, all yes vs. no), and single versus multicenter study. Given the large number of analyses planned, they will be used in an exploratory manner and the results interpreted with caution. Examining the effect of differences in experimental design will inform aspects of a future clinical trial.

In preclinical studies, construct validity refers to the extent an animal model corresponds to the clinical entity it is intended to represent [35]. Construct validity will be assessed in relation to the extent the experimental systems model the clinical entity of perioperative MI using a framework based on expert opinion (see Table 4). It will help determine whether the included studies enable reliable causal inference and generalization to a potential clinical study of MSCs for perioperative MI.

**Table 4:** Checklist of construct validity for preclinical perioperative myocardial infarction (PeriopMI)

Construct validity domain	Criteria from guidelines^a,b^	Specific application to PeriopMI	Justification	Yes/No
Animal Subjects	Matching model to age of patients in clinical setting	Middle aged to elderly animal model used	Incidence of PeriopMI increases over age 50; age >75 is an independent risk factor for PeriopMI [46]	
	Matching model to co-morbidities in clinical setting	Animal model has ≥ 1 co-morbidity risk factor for PeriopMI, either chronic or acute (e.g. atherosclerosis, diabetes, chronic kidney disease, hypotension, acute blood loss)	Co-morbidities listed are independent risk factors for PeriopMI [47]	
Outcome Measures	Matching of outcome measure to clinical setting	Late outcome measures performed (e.g. >3 weeks when scar formation and acute changes are complete)	A longer follow-up duration may reflect chronic effects of an acute therapy for PeriopMI	
Modeling of Disease	Matching model to human manifestation of disease	Model reflects elements of Type 1 MI (e.g. plaque rupture) and/or Type 2 (e.g. supply demand imbalance)	Clinical PeriopMI displays aspects of Type 1 and Type 2 MI [30, 42]	
		A pro-inflammatory state is reported	Clinical PeriopMI has a large inflammatory burden [19]	
Administration of Intervention	Treatment response along mechanistic pathway	Therapy given as a pretreatment (i.e. preventative) or within the first 48 h after anesthesia	Majority of PeriopMI occurs within the first 48 h after surgery	
Environment	Address confounds associated with setting, experimental setting	Post-operative analgesia provided	Inadeqaute post-operative analgesia increases systemic inflammation	

*Abbreviations*: *MI* myocardial infarction, *PeriopMI* perioperative myocardial infarction

^a^Recommendations to reduce threats to construct validity were identified by Henderson et al. [19]

^b^Construct validity criteria suggested by ≥40% of included guidelines included in checklist

### Strategy for data synthesis

Search results will be presented in a PRISMA study flow diagram [15]. Categorical variables will be summarized by frequencies/percentages, and continuous variables will be summarized by means and standard deviations or median and interquartile ranges, depending on data distribution.

Dichotomous endpoints (e.g., death) from each included study will be pooled and described as odds ratios and 95% confidence intervals. Results from outcomes with discrete data will be pooled, and meta-analysis will be performed with inverse variance random effects modeling. Continuous endpoints will be pooled using the ratio of weighted means method with inverse variance random effects modeling [36]. Ratio of means allows for pooling of outcomes expressed in different units and comparisons of effect sizes across interventions. As ratio of means is well suited for the small sample sizes of animal studies, and provides a result in a form similar to a risk ratio, we have chosen this method because of its simplified clinical interpretation. Statistical heterogeneity will be examined using *I* ^2^ tests with 95% uncertainty intervals [37]. Planned sensitivity analyses will examine heterogeneity of the primary outcome. These will be carried out according to risk of bias assessments. Selective outcome reporting will be assessed using the excess significance test (comparing the expected percentage of significant results vs. actual reported effects) [38]. An evaluation for the presence of publication bias will be conducted with funnel plot techniques and Egger’s regression test [39].

### Knowledge translation

Several knowledge users of the results of this systematic review have been identified. These include the Canadian Perioperative Anesthesia Clinical Trials (PACT) Group, a network of academic perioperative medicine researchers that develop team-based approaches to investigate perioperative clinical and basic science questions (www.canadianpact.ca). Our other knowledge users include the Canadian Council on Animal Care, Canadian Society for Atherosclerosis Thrombosis, and Vascular Biology (www.csatvb.ca) and the Stem Cell Foundation of Canada (www.stemcellfoundation.ca). Through these users, our research will reach key perioperative researchers, preclinical and translational scientists, and health professionals as well as the lay community.

This work will identify gaps in the current knowledge of MSC therapy of MI. Publication of our results will also identify potential future directions of MSC for MI research as they specifically relate to perioperative MI. Most importantly, the publication of key findings of the review and meta-analysis will directly inform a potential clinical trial.

## Discussion

This review proposes to systematically identify and summarize preclinical evidence that exists regarding MSC therapy in myocardial infarction models, using a rigorous methodology. We will assess the effect of MSC therapy on clinically important outcomes including cardiac function, infarct size, inflammation, and death.

In our pilot searches, we have identified two published preclinical reviews that investigated stem cells in both acute and chronic ischemia models [24, 25]. Our proposed preclinical review differs significantly from these studies in several respects. Both previous studies combined results from various stem cell types and were restricted to large animal models, whereas our review will focus on MSCs and consider both small and large animal models. The search strategies used in these papers identified 39 studies that used MSCs; however, based on our pilot search using a comprehensive strategy, we have identified approximately 200 studies to be included in our review. Most importantly, the data from these reviews included cell therapy for chronic heart failure (48% of studies); therapy of established chronic heart failure has little construct validity for the acute treatment of MI. Thus, our review will provide novel evidence to determine if a clinical study of MSCs for MI is warranted.

Given that less than 5% of high impact preclinical reports are clinically translated [40] and only 11% of clinically tested agents receive licensing, rigorous appraisal of preclinical data is needed prior to clinical testing of novel therapeutics. Historically, failed translation (preclinical to clinical) of specific therapies for stroke [41] and heart failure [42] could have been predicted by systematic reviews of animal data. Thus, our review is critical prior to conducting a resource intensive trial.

Furthermore, since the design of these preclinical studies wil include administration of anesthetic and disease induction that likely differs from spontaneous MI, this review also provides a unique opportunity to determine if MSCs may have efficacy in clinical perioperative MI. This question is of particular interest to our group as therapies that are effective in prevention of non-operative MI have failed to show benefit in perioperative MI [43–45] and there are currently few therapies for established perioperative MI. Given what is known about the mechanism of action of MSCs, they may be highly effective in the pro-inflammatory state that occurs with perioperative MI.

In summary, this review will be the first to provide an estimate of efficacy and safety of MSC therapy in preclinical models of MI. This will ultimately help determine whether sufficient evidence exists to support a first-in-human evaluation of MSC therapy for perioperative MI. Additionally, the results of this study will identify knowledge gaps and potential future areas of study in MI research.

## Additional file

Additional file 1: Description: representative search strategy. (DOCX 13 kb)

## Abbreviations

LVEF: Left ventricular ejection fraction
MI: Myocardial infarction
MSCs: Mesenchymal stromal cells
