# Supplementary material for: Assessment of safety and efficacy of mesenchymal stromal cell therapy in preclinical models of acute myocardial infarction: a systematic review protocol
Source: Syst Rev. 2017 Nov 7;6:226. doi: 10.1186/s13643-017-0601-9 (PMC5688817; doi:10.1186/s13643-017-0601-9)
Supplement: Additional file 1: — Description: representative search strategy. (DOCX 13 kb) [file 13643_2017_601_MOESM1_ESM.docx]

**Representative Search Strategy**

Database: Ovid MEDLINE(R) In-Process & Other Non-Indexed Citations and Ovid MEDLINE(R) <1946 to Present>

--------------------------------------------------------------------------------

1 Mesenchymal Stromal Cells/

2 Mesenchymal Stem Cell Transplantation/

3 Multipotent Stem Cells/

4 (MSC or MSCs or ADMSC or ADMSCs or BMDMSC or BMDMSCs).tw.

5 (mesenchymal adj5 (cell$ or stem or stromal or progenitor or multipotent or bone marrow or adipose or placenta$)).tw.

6 ((multipotent or multi-potent) adj (stroma$ cell$ or stem cell$ or progenitor cell$)).tw.

7 (marrow stroma$ adj2 cell$1).tw.

8 or/1-7

9 exp animal experimentation/ or exp models, animal/ or animals/ or mammals/ or vertebrates/ or exp fishes/ or exp amphibia/ or exp reptiles/ or exp birds/ or exp hyraxes/ or exp marsupialia/ or exp monotremata/ or exp scandentia/ or exp chiroptera/ or exp carnivora/ or exp cetacea/ or exp Xenarthra/ or exp elephants/ or exp insectivora/ or exp lagomorpha/ or exp rodentia/ or exp sirenia/ or exp Perissodactyla/ or primates/ or exp strepsirhini/ or haplorhini/ or exp tarsii/ or exp platyrrhini/ or catarrhini/ or exp cercopithecidae/ or gorilla gorilla/ or pan paniscus/ or pan troglodytes/ or exp pongo/ or exp hylobatidae/ or hominidae/

10 (animal$1 or chordata or vertebrate* or fish$2 or amphibian* or amphibium* or reptile$1 or bird$1 or mammal* or dog or dogs or canine$1 or cat or cats or hyrax* or marsupial* or monotrem* or scandentia or bat or bats or carnivor* or cetacea or edentata* or elephant* or insect or insects or insectivore or lagomorph* or rodent$2 or mouse or mice or murine or murinae or muridae or rat or rats or pig or pigs or piglet$1 or swine or rabbit$1 or sheep$1 or goat$1 or horse$1 or equus or cow or cows or cattle or calf or calves or bovine or sirenia or ungulate$1 or primate$1 or prosimian* or haplorhini* or tarsiiform* or simian*or platyrrhini or catarrhini or cercopithecidae or ape or apes or hylobatidae or hominid* or chimpanzee* or gorilla* or orangutan* or monkey or monkeys or ape or apes).tw.

11 (preclinic$ or pre clinic$).tw.

12 or/9-11

13 8 and 12

14 exp Myocardial Infarction/

15 ((myocardial or cardiac or heart) adj2 (infarct$ or attack$)).tw.

16 exp Heart Failure/

17 ((cardiac or heart) adj failure).tw.

18 Coronary Artery Disease/

19 coronary artery disease$.tw.

20 (coronary adj (arterioscleros$ or atheroscleros$)).tw.

21 ((myocard$ or card$) adj2 regeneration).tw.

22 (cardiac repair or myocard$ repair).tw.

23 Myocardial Reperfusion Injury/

24 ((card$ or myocard$) adj2 (ischem$ or ischaem$)).tw.

25 ((ischem$ adj2 reperfusion) and (heart or card$ or myocard$)).tw.

26 ((ischaem$ adj2 reperfusion) and (heart or card$ or myocard$)).tw.

27 (Reperfusion Injur$ adj2 (card$ or myocard$ or heart)).tw.

28 Myocardial Ischemia/

30 13 and 29

31 limit 30 to english language
